# Alpha retinal ganglion cells in pigmented mice retina: number and distribution

**DOI:** 10.3389/fnana.2022.1054849

**Published:** 2022-12-01

**Authors:** Alejandro Gallego-Ortega, María Norte-Muñoz, Johnny Di Pierdomenico, Marcelino Avilés-Trigueros, Pedro de la Villa, Francisco Javier Valiente-Soriano, Manuel Vidal-Sanz

**Affiliations:** ^1^Department of Ophthalmology, Instituto Murciano de Investigación Biosanitaria-Virgen de la Arrixaca (IMIB-Arrixaca), Universidad de Murcia, Murcia, Spain; ^2^Department of Systems Biology, Laboratory of Visual Neurophysiology, School of Medicine and Health Sciences, University of Alcalá, Alcalá de Henares, Spain; ^3^Instituto Ramón y Cajal de Investigación Sanitaria (IRYCIS), Hospital Ramón y Cajal, Madrid, Spain

**Keywords:** alpha retinal ganglion cells, intrinsically photosensitive retinal ganglion cells, osteopontin (OPN), melanopsin (OPN4), ON sustained alpha retinal ganglion cells, ON transient alpha retinal ganglion cells, OFF sustained alpha retinal ganglion cells, OFF transient alpha retinal ganglion cells

## Abstract

**Purpose:** To identify and characterize numerically and topographically the population of alpha retinal ganglion cells (αRGCs) and their subtypes, the sustained-response ON-center αRGCs (ONs-αRGCs), which correspond to the type 4 intrinsically photosensitive RGCs (M4-ipRGCs), the transient-response ON-center αRGCs (ONt-αRGCs), the sustained-response OFF-center αRGCs (OFFs-αRGCs), and the transient-response OFF-center αRGCs (OFFt-αRGCs) in the adult pigmented mouse retina.

**Methods:** The αRGC population and its subtypes were studied in flat-mounted retinas and radial sections immunodetected against non-phosphorylated high molecular weight neurofilament subunit (SMI-32) or osteopontin (OPN), two αRGCs pan-markers; Calbindin, expressed in ONs-αRGCs, and amacrines; T-box transcription factor T-brain 2 (Tbr2), a key transcriptional regulator for ipRGC development and maintenance, expressed in ipRGCs and GABA-displaced amacrine cells; OPN4, an anti-melanopsin antibody; or Brn3a and Brn3c, markers of RGCs. The total population of RGCs was counted automatically and αRGCs and its subtypes were counted manually, and color-coded neighborhood maps were used for their topographical representation.

**Results:** The total mean number of αRGCs per retina is 2,252 ± 306 SMI32^+^αRGCs and 2,315 ± 175 OPN^+^αRGCs (*n* = 10), representing 5.08% and 5.22% of the total number of RGCs traced from the optic nerve, respectively. αRGCs are distributed throughout the retina, showing a higher density in the temporal hemiretina. ONs-αRGCs represent ≈36% [841 ± 110 cells (*n* = 10)] of all αRGCs and are located throughout the retina, with the highest density in the temporal region. ONt-αRGCs represent ≈34% [797 ± 146 cells (*n* = 10)] of all αRGCs and are mainly located in the central retinal region. OFF-αRGCs represent the remaining 32% of total αRGCs and are divided equally between OFFs-αRGCs and OFFt-αRGCs [363 ± 50 cells (*n* = 10) and 376 ± 36 cells (*n* = 10), respectively]. OFFs-αRGCs are mainly located in the supero-temporal peripheral region of the retina and OFFt-αRGCs in the mid-peripheral region of the retina, especially in the infero-temporal region.

**Conclusions:** The combination of specific antibodies is a useful tool to identify and study αRGCs and their subtypes. αRGCs are distributed throughout the retina presenting higher density in the temporal area. The sustained ON and OFF response subtypes are mainly located in the periphery while the transient ON and OFF response subtypes are found in the central regions of the retina.

## Introduction

Retinal ganglion cells (RGCs) are located in the innermost layer of the retina and are responsible for projecting elaborated computations of the light signals that arise from the photoreceptors to regions of the brain for processing (Bray et al., 1987; Aguayo et al., 1990). Since they were drawn and characterized by Santiago Ramón y Cajal in 1892 (Ramón Y Cajal, 1892), many studies have classified these cells according to their morphology, size, functionality, or molecular attributes (Jeon et al., 1998; Sanes and Masland, 2015; Baden et al., 2016; Krieger et al., 2017) with the latest studies in mice pointing to over 40 different RGC types (Bae et al., 2018; Rheaume et al., 2018; Tran et al., 2019; Goetz et al., 2022). In the rat retina, three main types of RGCs were described according to their morphology and the size of their soma and dendrites: large (A), small (B), and medium (C) (Huxlin and Goodchild, 1997). Of these, the most studied in the literature are type A, also called alphaRGCs (αRGCs) which were described in detail for the first time in the cat retina (Cleland and Levick, 1974a,b; Cleland et al., 1975). These cells are recognizable by their large cell bodies, robust dendrites and axons, large monostratified dendritic branches with different strata of the inner plexiform layer (IPL), high levels of neurofilament proteins, and large receptive fields (Dreher et al., 1985; Peichl, 1989). In addition to their morphological characteristics, these αRGCs share common physiological properties, such as short response latency and fast-conducting axons that allow them to be the first to respond to a new stimulus (Cleland et al., 1975). αRGCs can be functionally subdivided into ON and OFF centers that respond to increases (ON) and decreases (OFF) in light intensity within the center of their receptive field, which also corresponds to the anatomical layering of the dendrites within the IPL (Nelson et al., 1978). Moreover, these responses have been further subdivided into sustained or transient with a corresponding distribution of their dendrites within the sustained or transient IPL regions (Euler et al., 2014).

To date, four different types of αRGCs have been described in mice according to their receptive fields and signal patterns and named based on their mono-stratification with the IPL from the inner to the outer part, these are: ON-sustained (ONs-αRGCs), ON-transient (ONt-αRGCs), OFF-sustained (OFFs-αRGCs) and OFF-transient (OFFt-αRGCs; Krieger et al., 2017; Wang et al., 2021). Detailed electrophysiological characterization of the responses of αRGCs in adult mice was carried out by Pang et al. (2003) and this study yielded three main types including: OFFs-αRGCs, OFFt-αRGCs, and ON-αRGCs without further distinction into sustained or transient types, although the authors state that all the 28 ON-αRGCs cells examined had a similar response (illustrated in their Figures 1E, 2) which appears to be of the sustained type. This study, however, did not analyze αRGC topographic distribution (Pang et al., 2003). The morphological study by Hong et al. (2011) described the soma, dendritic field distribution within the inner plexiform layer, and axonal projection of several αRGC types, including; X3 (presumably OFFs-αRGCs), X5 (presumably OFFt-αRGCs), and X7 (presumably ON-αRGCs without a clear distinction between transient or sustained response). This work showed details about the distribution of their dendritic field and soma size and related these values to their axonal arborization within the superior colliculus, but did not analyze their topographical distribution over the retina (Hong et al., 2011). The study of Schmidt et al. (2014) documented that ON-αRGCs corresponded to one of the types of intrinsically photosensitive RGCs expressing melanopsin, the type M4 (Ecker et al., 2010; Estevez et al., 2012), and suggested that this type (ON-αRGCs/M4), as defined by the expression of the alfa RGC marker (SMI32) and melanopsin (OPN4), had a total number of approximately 856 RGCs that were distributed across all retinal quadrants (illustrated in their Figure 1D). The discovery of the fourth type of αRGCs, the ONt-αRGCs was done by the work of Krieger et al. (2017) using a combination of molecular, *in vitro* electrophysiological recordings, as well as detailed morphological and immunohistochemical analysis. Using these techniques Krieger et al. (2017) characterized the ONt-αRGCs, described the distinct immunohistochemical signature of each of the four αRGC types and analyzed the density recovery profile of each of these αRGCs suggesting that these were different types with a distinct mosaic distribution within the retina, although their topographical distribution within the retina was not addressed (Krieger et al., 2017). Furthermore, not all αRGCs appear to be of large soma size with large dendritic fields and arborizations, as smaller alpha cells have been recently described in adult rats (Tan et al., 2022) and mice (Baden et al., 2016). In adult rats, a small cluster of αRGCs, distributed throughout the retina, but with soma and dendritic field sizes not matching classic morphological features of αRGCs (Peichl, 1989, 1991) was identified using SMI32 antibodies (Tan et al., 2022). In adult mice, mini-alpha RGCs have been described (Baden et al., 2016; Ran et al., 2020; Goetz et al., 2022), and these have smaller somatic and dendritic field sizes and show transient responses of the OFF-αRGCs or ON-αRGCs types, but are not detectable with SMI32 antibodies (Baden et al., 2016), and thus were not considered in our present study.

Regarding the spatial localization of RGCs, early work describing αRGCs in the cat, rat, and rabbit retina (Wassle et al., 1975; Provis, 1979; Peichl et al., 1987; Peichl, 1991) showed that these cells were distributed throughout the retina, showing a higher density in the center-temporal region with a gradual decrease in density toward the periphery, showing that their density changes paralleled changes in total ganglion cell density. This non-uniform topographical distribution and the different sizes of their receptive fields, different from the topographical patterns of other mouse RGCs, indicate that visual space is heterogeneously sampled by distinct retinal processing circuits organized by distinguished populations of RGCs that explore and scan specific visual areas (Bleckert et al., 2014). Indeed, previous studies have shown that the dendritic arbors of the ON- and OFF-αRGCs sustained response subtypes have an asymmetric behavior depending on their spatial location, with a negative correlation of size in the naso-temporal axis with smaller cells located in the temporal region and greater arborization in the more peripheral cells (Bleckert et al., 2014; Reinhard et al., 2019). However, there is no clear consensus on the organization of the dendritic fields of OFFt-αRGCs, as Wang et al. (2021) observed a more homogeneous distribution of their dendritic fields, regardless of their spatial location on the retina, but Warwick et al. (2018) reported a negative correlation of dendritic field size along the dorsal-ventral axis for OFFt-αRGCs, which showed a smaller size in the ventral region, as well as a change in the functional response of OFFt-αRGCs from transient to sustained when stimulated along the ventral-dorsal axis, which is related to the input of the primary rod pathway through amacrine II cells (Warwick et al., 2018). This change in response may reflect differences between the upper and lower visual fields (Warwick et al., 2018), and this may be related to the ethological optimal response of the OFFt-αRGCs which are sensitive to looming approaching the sky at varying light intensities, in the upper visual field which is imaged by the ventral retina (Warwick et al., 2018; Wang et al., 2021).

**Figure 1:**
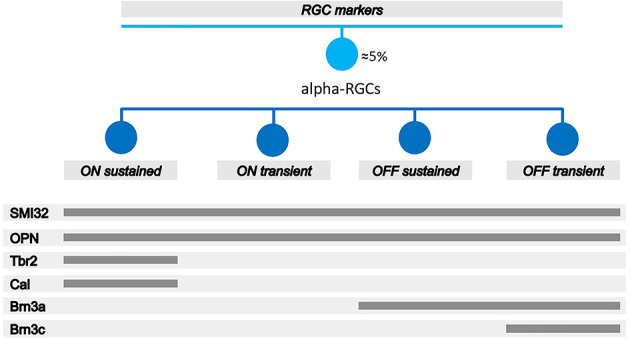
Schematic diagram of the different antibodies to identify αRGCs and its subtypes by immunohistochemistry. SMI32 and OPN antibodies are pan-markers of αRGCs. The combination of SMI32^+^ or OPN^+^ and Calbindin^+^ or Tbr2^+^ antibodies are used to identify ONs-αRGCs; OPN^+^, Brn3a^−^, and Tbr2^−^ antibodies are used to identify ONt-αRGCs; SMI32^+^ or OPN^+^ and Brn3a^+^ and Brn3c^−^ are used to identify OFFs-αRGCs; and SMI32^+^ or OPN^+^ and Brn3a^+^ and Brn3c^+^ are used to identify OFFt-αRGCs. Figure adapted and modified from Krieger et al. (2017).

αRGCs can be detected using transgenic mice in which αRGCs express Cre recombinase which can be detected by fluorescence (Krieger et al., 2017) or by immunohistochemistry using the antibody against osteopontin (OPN), a secreted phosphoprotein (Spp1; Duan et al., 2015; Mayer et al., 2018; Honda et al., 2019; Gallego-Ortega et al., 2021) or with SMI32, an antibody that recognizes the nonphosphorylated epitope on the medium- and high-molecular-weight subunits of neurofilament proteins (Schmidt et al., 2014; Lee and Schmidt, 2018; Wang et al., 2021; Tan et al., 2022; Figure 1). Each subtype of αRGCs can be immunodetected using a combination of antibodies according to the expression of the Pou4f (Brn3) gene family. ONs-αRGCs are Brn3a^−^Brn3b^+^Brn3c^−^Calbindin^+^, ONt-αRGCs are Brn3a^−^Brn3b^+^Brn3c^−^Calbindin^−^, OFF-s-αRGCs are Brn3a^+^Brn3b^+^Brn3c^−^ and OFFt-αRGCs are Brn3a^+^Brn3b^+^Brn3c^+^ (Krieger et al., 2017). It has been documented that ONs-αRGCs are a type of intrinsically photosensitive ganglion cells, the M4-ipRGCs (Ecker et al., 2010; Schmidt et al., 2014; Sonoda et al., 2020). However, these cells are not easy to identify with the classical OPN4 antibody against melanopsin, which identifies M1, M2, and M3 ipRGCs, as they do not express sufficient melanopsin photopigment (Vidal-Villegas et al., 2019, 2021a,b), although their functional dependence on pigment has been characterized (Estevez et al., 2012), which has led to a lack of global consensus on the overall identification of the total M4-ipRGC population and the number of cells per retina in rodents. It has recently been reported that cells expressing the photopigment also express the transcription factor T brain 2 (Tbr2), which is crucial for the maintenance of these cells (Berg et al., 2019; Tran et al., 2019; Chen et al., 2021). Therefore, other authors have used the colocalization of OPN, as a marker for αRGCs, and Tbr2, as a reliable marker of M4-ipRGCs (Gallego-Ortega et al., 2021). In addition, other authors have characterized the presence of calbindin expressed in ONs-αRGCs (in addition to the labeling of a subpopulation of amacrine cells; Krieger et al., 2017; Sonoda et al., 2020) which, when colocalized with an αRGC pan-marker, allows for reliable detection of ONs-αRGCs. Previous studies have documented that these M4-ipRGCs (ONs-αRGCs) are more resistant to retinal injury (El-Danaf and Huberman, 2015; Ou et al., 2016; Tran et al., 2019), due to their ability to alter their synaptic connectivity pattern after injury to promote survival (Della Santina et al., 2021). However, there is not a complete consensus on this characteristic, as in other studies, M4-ipRGCs have not been more resistant than the rest of the RGCs (Gallego-Ortega et al., 2021; Gao et al., 2022), reviewed in Tran et al. (2019).

In the present study, we aimed to further characterize αRGCs and their subtypes, by counting the total number of αRGCs using immunohistochemical and image analysis techniques, and by examining their topological distribution patterns in the retina of adult pigmented mice.

## Material and Methods

### Animal handling and experimental design

Animal care and experimental procedures were approved by the Ethical and Animal Studies Committee of the University of Murcia (UM; Spain; Codes: A13171103, A13170110, and A13170111) and followed the Spanish and European Union regulations for the use of animals in research (Council Directive 86/609/EEC) and the ARVO statement for the use of animals in ophthalmic and vision research. In this work, we have used pigmented C57BL/6J mice (20–24 g) provided by the animal facility of the UM and housed in temperature-controlled rooms with 12-h light/dark cycles and with food and water provided *ad libitum*. To perform surgery on the tracing of the RGCs from the optic nerve (see below), mice were anesthetized with a mixture of intraperitoneal ketamine (60 mg/kg body weight, Ketolar; Pfizer, Alcobendas, Madrid, Spain) and xylazine (10 mg/kg Rompun; Bayer, Kiel, Germany).

A total of 37 pigmented mice were used in this study. To study the entire population of RGCs in the pigmented mouse retina, three mice (six retinas) were used to detect the total population of RGCs, both traced from the optic nerve and detected by immunohistochemistry (see below). To study the population and distribution of αRGCs and their subtypes, 30 mice (60 retinas) were used for the study of the retina in whole mounts and four additional mice (eight retinas) were used for the study of the retina in radial sections.

### Tracing the whole RGC population

In anesthetized mice, the ONs were exposed intra-orbitally following previously described methods that are standard in our laboratory (Avilés-Trigueros et al., 2000; Vidal-Sanz et al., 2002, 2015, 2017; Nadal-Nicolás et al., 2015a,b). A gelatin sponge (Spongostan Film, Ferrosan A/S, Denmark) impregnated with the tracer hydroxyl stilbamidine methanesulfonate (OHSt, Molecular Probes, Leiden, The Netherlands), a Fluorogold analog, [10% dimethyl-sulfoxide (DMSO) in 0.9% saline], was applied ~1–2 mm around the ocular portion of the optic nerve as described (Nadal-Nicolás et al., 2015a,b). The eye fundus was inspected after the procedure and mice were sacrificed 3 days after tracing.

### Tissue processing

All mice were sacrificed at the same time range (10.00–11.00 am) to avoid diurnal fluctuation in protein expression, by intraperitoneal lethal injection of pentobarbital (Vetoquinol Dolethal, Especialidades Veterinarias, S.A., Alcobendas, Madrid, Spain). Mice were then transcardially perfused with saline and 4% paraformaldehyde (PFA) in 0.1 M phosphate buffer (pH 7.4; Nadal-Nicolás et al., 2009; Valiente-Soriano et al., 2014). The eyes were enucleated and postfixed for a further 1 h in 4% PFA and divided for retinal study in flat mounts or radial sections. For the study of the flat mounts, the retinas were dissected by making four radial cuts, with the deepest superior cut to maintain the orientation of the retina at each moment, following standard protocols in our laboratory (Nadal-Nicolás et al., 2012; Valiente-Soriano et al., 2014). For the study of radial sections, the cornea and crystalline lens were discarded while maintaining the optical cups which were cryoprotected in increasing concentrations of sucrose before being embedded in an optimal cutting temperature (OCT) compound (Sakura Finetek, Torrance, USA) for cryostat sectioning (16 μm; Nadal-Nicolás et al., 2015b).

### Immunohistofluorescence

To study the total RGC population, αRGCs and their subtypes, both in flat retinas and in radial sections, immunohistochemical techniques were performed as described previously (Valiente-Soriano et al., 2014; Gallego-Ortega et al., 2020). Briefly, retinas previously permeabilized in 0.5% Triton X100 PBS were incubated overnight with primary antibodies diluted in blocking buffer (PBS, 2% normal donkey or goat serum, 2% Triton X100). The retinas were then washed in PBS 0.5% Triton X100 and incubated at room temperature for 2 h with appropriate secondary antibodies (1:500 concentration) diluted in PBS 0.5% Triton X100. Finally, the retinas were washed in PBS and mounted vitreous side up on slides and covered with an antifading solution (Galindo-Romero et al., 2013b; Valiente-Soriano et al., 2014).

To detect the total population of RGCs, the RNA-binding protein with multiple splicing (RBPMs) antibody was used (Rodriguez et al., 2014). To detect the αRGC population, SMI32 and OPN antibodies were used (Figure 1; Sonoda et al., 2020; Tan et al., 2022). For the study of the αRGCs subtypes, ONs-αRGCs, ONt-αRGCs, OFFs-αRGCs, and OFFt-αRGCs, different combinations of antibodies detailed in Figure 1 were used (Krieger et al., 2017; Gallego-Ortega et al., 2021; Tan et al., 2022). Previous studies have described that ONs-αRGCs express Calbindin (Krieger et al., 2017) and Tbr2 (Chen et al., 2021), so their combination with the αRGCs panmarkers SMI32 or OPN antibodies, which identify the total αRGC population, allows us to immunodetect these cells (Figure 1; Gallego-Ortega et al., 2021). Moreover, because ONs-αRGCs correspond to M4, one of the ipRGC types, we also investigated if ONs-αRGCs could be detected with OPN4 melanopsin antibody (Gallego-Ortega et al., 2021; Vidal-Villegas et al., 2021a). To identify the ONt-αRGC population, we used the combination of the αRGCs panmarkers SMI32 or OPN positive signal with a negative signal of Tbr2 (only expressed in ONs-αRGC; Chen et al., 2021) and Brn3a (only expressed in OFF-αRGCs; Krieger et al., 2017; Figure 1). To identify OFF-αRGCs, we used the brn3a antibody in combination with Brn3c, negative for OFFs-αRGCs and positive for OFFt-αRGCs (Krieger et al., 2017), and with the αRGCs panmarkers SMI32 or OPN (Figure 1). All primary and secondary antibodies used in this work are listed in Table 1.

**Table 1:** Primary and secondary antibodies used in this work.

	**Antibody**	**Reference**	**Dilution**
Primaries	Mouse anti Brn3a	MAB1585 Millipore	1:500
	Goat anti Osteopontin	AF808 Biotech	1:1,000
	Rabbit anti Tbr2	AB23345 Abcam	1:1,000
	Guinea pig anti Calbindin D28k	214 005 Synaptic Systems	1:500
	Mouse anti Neurofilament H (NF-H), Nonphosphorylated (SMI32)	BioLegend 801702	1:2,000
	Rabbit anti OPN4	AB-N39 Advanced Targeting Systems	1:1,000
	Rabbit anti RBPMS	Invitrogen PA595647	1:500
	Guinea pig anti Brn3a	411004 Synaptic Systems	1:500
	Mouse anti Brn3c	AB58128 Abcam	1:500
Secondaries	Goat anti mouse Igg1 Alexa 555	A21127 Molecular Probes Thermo-Fisher	1:500
	Donkey anti rabbit Alexa 488	A21206 Molecular Probes Thermo-Fisher	1:500
	Donkey anti goat Alexa 647	A32849 Molecular Probes Thermo-Fisher	1:500

### Image analysis

Flat mounts and radial sections previously immunostained were analyzed and photographed with an epifluorescence microscope (Leica DM6-B; Leica Microsystems, Wetzlar, Germany) as previously described (Gallego-Ortega et al., 2021). In brief, in reconstructions of retinal flat mounts and radial retinal sections, multiple frames were acquired for each filter in a raster scan pattern (×20) contiguously, side-by-side with no overlap or spacing between images. Individual images were focused on before acquisition and obtained with the same focus for each of the specific filters used. To acquire retinal magnifications, ×40 and ×60 objectives were used (Gallego-Ortega et al., 2021).

### Quantification and co-expression analysis

To determine in flat-mounted retinas the total numbers of RGCs traced retrogradely with OHSt from the optic nerve or immunolabeled with RBPMs^+^RGCs, standard computer routines developed in our laboratory were used (Nadal-Nicolás et al., 2015a; Vidal-Sanz et al., 2015, 2017). For the study of the population of αRGCs immunodetected against SMI32 or OPN, these were manually dotted on each retinal photomontage, and then the total number of dots per retina was quantified using Image Pro Plus software (IPP 5.1 for Windows^®^; Media Cybernetics, Silver Spring, MD, USA) as previously described (Gallego-Ortega et al., 2021). To investigate retinal co-expression of SMI32 and OPN in flat-mounted retinas with no trace of blood to avoid artifacts or undesirable markings (mice were perfused transcardially with saline and PFA, see the previous section), individual frames from eight different retinal areas, covering the central and peripheral region of the supero-temporal, supero-nasal, infero-temporal, and infero-nasal quadrants, were superimposed and merged with Adobe Photoshop (21.2.1, Adobe System Incorporated, USA). To assess the proportion of αRGCs expressing SMI32, OPN, or both, an expert investigator dotted manually each RGC that was clearly stained with each antibody, and RGCs that changed color when images were merged were considered positive for both antibodies. The same protocol was used to identify each αRGC subtype. OPN^+^ or SMI32^+^, and Tbr2^+^ or Calbindin^+^ cells were considered ONs-αRGCs, OPN^+^Brn3a^−^Tbr2^−^ cells were considered ONt-αRGCs, OPN^+^Brn3a^+^Brn3c^−^ or SMI32^+^Brn3a^+^Brn3c^−^ cells were considered OFFs-αRGCs and OPN^+^Brn3a^+^Brn3c^+^ or SMI32^+^Brn3a^+^Brn3c^+^ cells were considered OFFt-αRGCs (Figure 1).

To study αRGCs in radial sections, three transverse sections in the nasotemporal axis containing the optic disc from each retina were selected. From each section, eight magnifications (four from the nasal and four from the temporal, 570 × 570 μm) were acquired at approximately 25%, 50%, 75%, and 95% distance from the optic disc towards the periphery as previously described (Di Pierdomenico et al., 2018) and the same protocol used in flat-mounted retinas were used to dot manually αRGCs and all their subtypes.

### Topographical distributions

The topographic distribution of the entire population of αRGCs and their subtypes were studied by neighbor maps using manually dotted photomontages using previously described methods (Galindo-Romero et al., 2013a; Valiente-Soriano et al., 2014). All maps were plotted using SigmaPlot (SigmaPlot11.0 for Windows; Systat Software, Inc., Richmond, CA, USA) using a color code.

## Results

### SMI32 and OPN as markers of αRGCs

**Figure 2:**
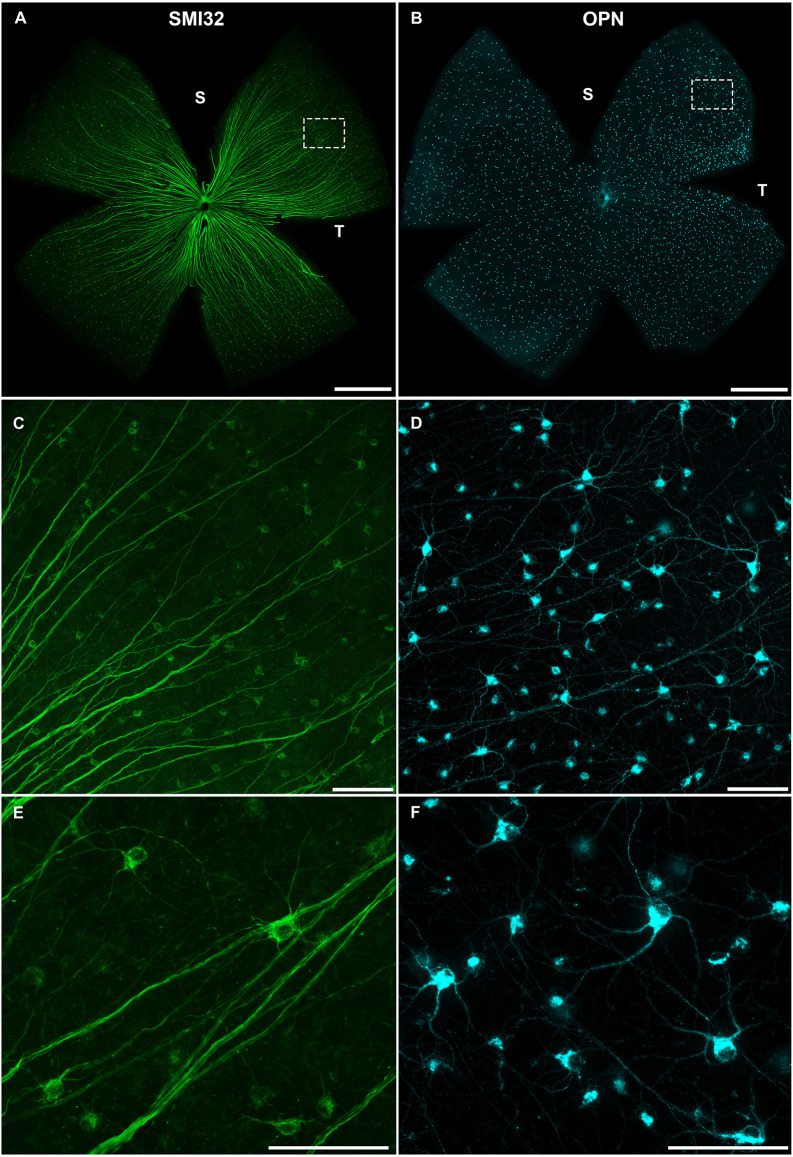
Representative images of αRGCs immunodetected in flat-mounted retinas against SMI32 or OPN. Photomontages of representative retinas immunodetected against SMI32 **(A)** or OPN **(B)**, two pan-markers of αRGCs. 20× **(C,D)** and 40× **(E,F)** magnifications of flat-mounted retina immunodetected with SMI32 or OPN showing whole-body labeling of stained αRGCs, including soma, proximal dendrites, and axons. Scale bar in **(A,B)**: 1 mm. Scale bar in **(C–F)**: 100 μm. S, Superior; T, Temporal.

**Figure 3:**
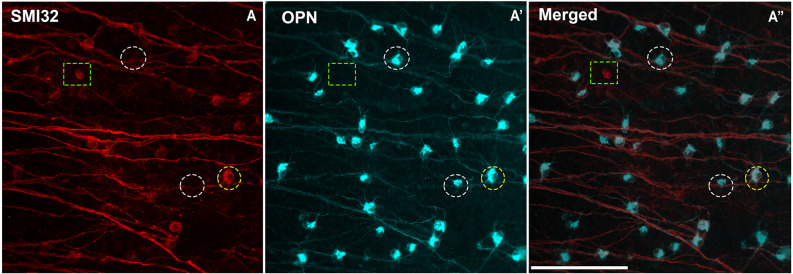
Colocalization between the two panmarkers of αRGCs: SMI32 and OPN. **(A–A”)** Representative micrographs from the temporal region of the mouse retina immunolabeled with OPN and SMI32. **(A)** Expression of SMI32 (in red) is located in αRGC axon, dendrites, and soma. **(A’)** Expression of the OPN (in blue) is located weakly in axons and dendrites but strongly in the somas of αRGCs. **(A”)** Superimposed and merged image with the SMI32 (in red) and OPN (in blue). Discontinuous white circles in **(A–A”)** represent a SMI32^−^OPN^+^αRGC, discontinuous green rectangles in **(A–A”)** represent a SMI32^+^OPN^−^ αRGC and discontinuous yellow circles in **(A,A”)** represent SMI32^+^OPN^+^αRGC. Scale bar: 100 μm.

Previous studies have indicated the expression of both SMI32 and OPN in αRGCs, identifying them as effective panmarkers for these cells (Duan et al., 2015; Krieger et al., 2017; Lee and Schmidt, 2018; Sonoda et al., 2020; Tan et al., 2022). In this study, we have used anti-OPN and anti-SMI32 antibodies to identify αRGCs and to analyze its distribution in the adult pigmented mouse retina (Figure 2). As shown in panels A, C, and E of Figure 2, the SMI32 antibody labels the entire cell body of αRGCs, including somas, dendrites, and axons. Although, the labeling is very specific and precise, the accumulation of labeled axons near the optic nerve head makes it problematic to locate their somas in this part of the retina (Figures 2, 3). In comparison, labeling of αRGCs with OPN antibody strongly labels somas and dendritic arbors, and lightly labels axons (Figures 2D,F, and 3), making it easier to identify αRGCs across the entire retinal surface (Figure 2B).

The colocalization study of the two αRGCs panmarkers, SMI32, and OPN, is shown in Figure 3 and Table 2. The results show that both markers colocalized in most instances but it was not homogeneous throughout the retina, with peripheral regions showing a higher percentage of colocalization (89.7%) than central regions (82.3%). However, no such differences were found between the superior (86.1%), inferior (85.7%), temporal (86.2%), or nasal (85.7%) regions (Table 2). This is due to the difficulty in perceiving the soma of SMI32-labeled RGCs in the central retinal regions, as the marker is highly immunoreactive in the nerve fibers of the retinal ganglion cells and does not allow for detecting the somas underneath these nerve fibers (Tan et al., 2022). However, the OPN marker was not as immunoreactive in the nerve fibers, so we could see the cell bodies and primary dendrites of αRGCs (Figure 3).

**Table 2:** Study of the percentage of RGCs expressing SMI32^+^OPN^+^, SMI32^+^OPN^−^ or SMI32^−^OPN^+^ in each of the retinal regions studied.

	**SMI32^+^OPN^+^**	**SMI32^+^OPN^−^**	**SMI32^−^OPN^+^**
STP	96.7 ± 1.2	1.6 ± 1.1	1.7 ± 1.4
STC	76.6 ± 7.4	4.3 ± 0.6	19.1 ± 14.2
ITC	82.2 ± 1.9	2.4 ± 2.1	15.4 ± 1.6
ITP	89.4 ± 4.9	9.6 ± 9.4	1.0 ± 0.4
SNP	83.8 ± 3.4	10.7 ± 4.9	5.5 ± 1.9
SNC	87.2 ± 7.2	1.8 ± 1.5	11.0 ± 12.9
INC	83.0 ± 6.2	7.0 ± 6.1	10.1 ± 6.4
INP	88.8 ± 4.7	9.1 ± 7.2	2.1 ± 2.2
Total average	88.1 ± 5.1	5.7 ± 3.5	6.2 ± 3.4

STP, supero-temporal periphery; STC, supero-temporal central; ITC, infero-temporal central; ITP, infero-temporal periphery; SNP, supero-nasal periphery; SNC, supero-nasal central; INC, infero-nasal central; INP, infero-nasal periphery.

**Figure 4:**
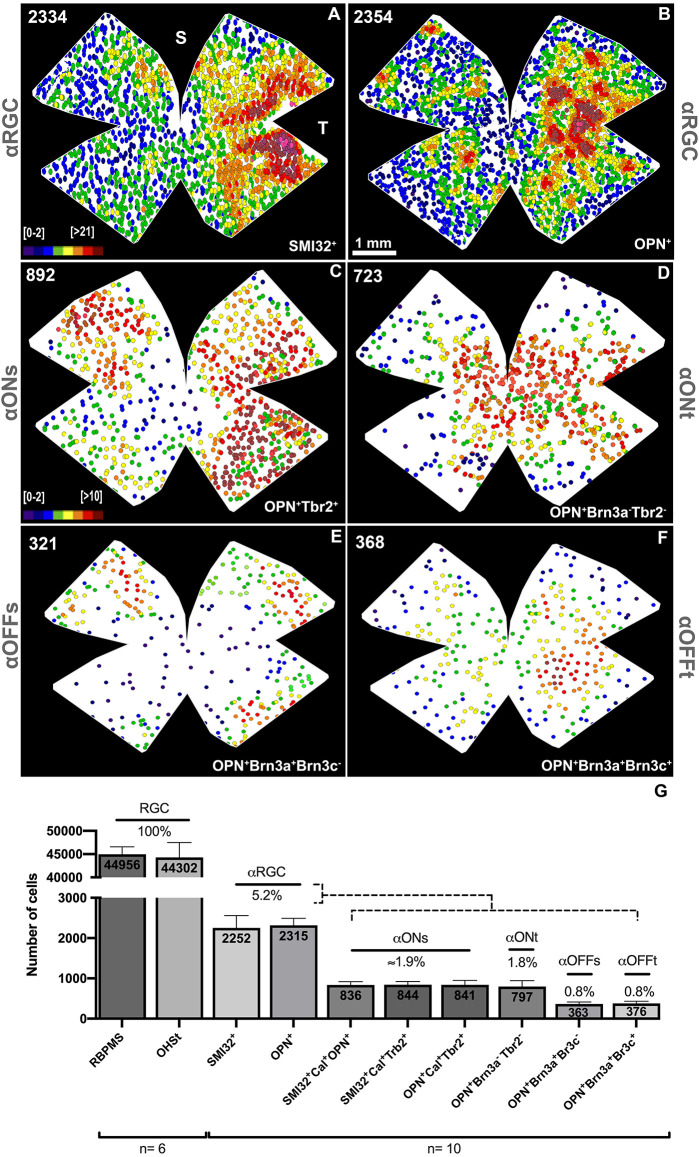
Maps of the topographical distribution of αRGCs and its subtypes. Representative neighbor maps of the retinal distribution of the SMI32^+^αRGCs **(A)**, OPN^+^αRGCs **(B)** and its subtypes ONs-αRGCs **(C)**, ONt-αRGCs **(D)**, and Off-αRGCs **(E)**. **(F)** Histogram showing the proportion of αRGC subtypes that constitute the total population of αRGC. **(G)** Bar histogram showing the mean of total RGCs retrogradely traced with OHSt from the optic nerve or immunodetected with RBPMs; αRGCs immunodetected against SMI32 or OPN; ONs-αRGCs (SMI32^+^Cal^+^OPN^+^, SMI32^+^Cal^+^Tbr2^+^, and OPN^+^Cal^+^Tbr2^+^); ONt-αRGCs (OPN^+^Brn3a^−^Tbr2^−^); OFFs-αRGCs (OPN^+^Brn3a^+^Brn3c^−^) and OFFt-αRGCs (OPN^+^Brn3a^+^Brn3c^+^). The percentages of each cell type are represented concerning the total number of RGCs retrogradely traced with OHSt. The color scale goes from 0 (purple) to 21 or more (red) **(A,B)** or 10 or more (red) **(C–F)** neighbors in a radius of 0.165 mm. The number in the superior left corner **(A–F)** reflects the total number of RGCs counted in that retina. S, Superior; T, Temporal. Scale bar: 100 μm.

The total number of αRGCs detected with both antibodies was very similar; 2,252 ± 306 SMI32^+^αRGCs vs. 2,315 ± 175 OPN^+^αRGCs, representing approximately 5.2% of the total number of RGCs labeled with OHSt applied around the uninjured optic nerve head (44,302 ± 3,197, *n* = 6) or immunodetected with RBPMs (44,956 ± 1,600, *n* = 6; Figure 4). The topographic maps of the SMI32^+^αRGCs and the OPN^+^αRGCs show an identical distribution. The αRGCs are distributed throughout the retina, from the center to the periphery, with a higher density in the temporal hemiretina (Figures 4A,B).

### Identification of αRGCs subtypes

#### ONsustained-αRGCs

To study ONs-αRGCs used two different protocols: (i) a combination of SMI32 or OPN antibodies with calbindin, which is expressed in the RGC layer, by amacrine and sustained ON-αRGCs (Berg et al., 2019; Tran et al., 2019; Chen et al., 2021); and (ii) the combination of SMI32 or OPN antibodies with Tbr2, which is a transcription factor that is expressed in all ipRGCs (Berg et al., 2019; Tran et al., 2019; Chen et al., 2021) and in some amacrine cells.

**Figure 5:**
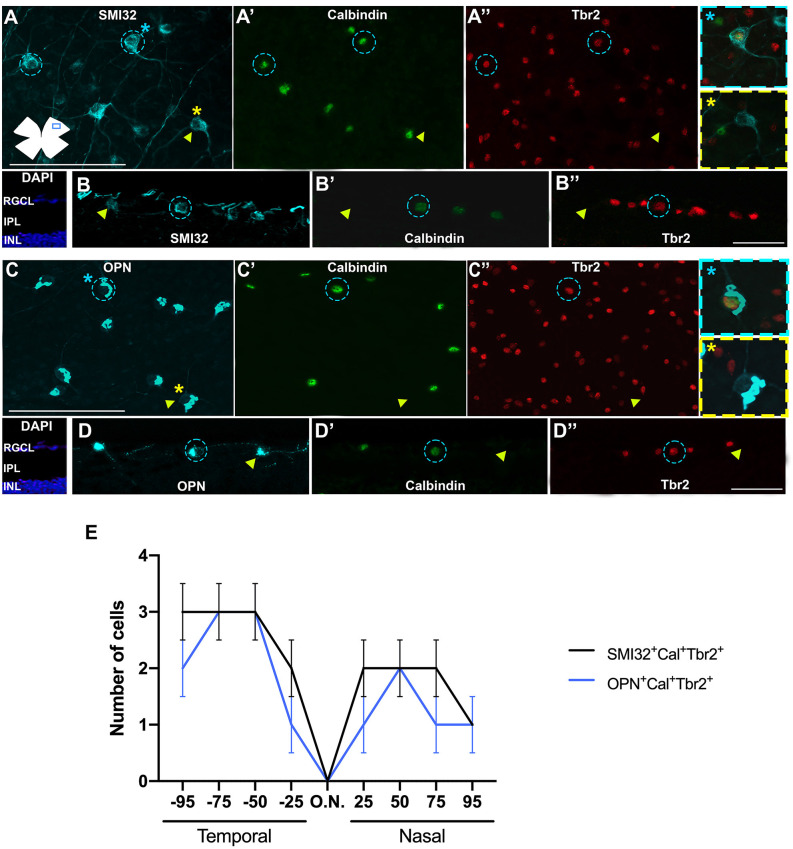
Immunohistochemical detection of ONs-αRGCs. Photomicrographs of flat-mounted retinas **(A–A”,C–C”)** or radial retinal sections **(B–B”,D–D”)** immunodetected against the αRGC pan-marker SMI32 **(A)** or OPN **(C)** in blue, Calbindin **(A’,C’)** in green and Tbr2 **(A”,C”)** in red in the same area of the retina. Note that calbindin and Tbr2 label the cell nuclei while SMI32, and OPN mark the cytoplasm of the soma and proximal axons. ONs-αRGCs were detected as SMI32^+^Cal^+^Tbr2^+^ or OPN^+^Cal^+^Tbr2^+^. **(E)** Graph illustrating manual counting of SMI32^+^Cal^+^Tbr2^+^ (black line) or OPN^+^Cal^+^Tbr2^+^ (blue line) in different regions of the retina, from 25-50-75-90% temporal to 25-50-75-90% nasal. In panels **(A–D”)**, blue circles represent cells positive for all three markers (SMI32^+^ or OPN^+^ and Calbindin^+^ and Tbr2^+^) merged in the panel with a blue asterisk and yellow arrowheads represent cells positive for the RGC pan-marker (SMI32^+^ or OPN^+^) but negative for Calbindin or Tbr2 merged in the panel with a yellow asterisk. Scale bar: 100 μm.

Immunohistochemical studies on both flat mounts and radial sections show that ONs-αRGCs are easily detectable with both protocols, indicating that this αRGC expresses all these proteins (SMI32, OPN, Calbindin, and Tbr2) and can be identified using SMI32 or OPN to locate αRGCs and Cal or Tbr2 which is specific for ONs-αRGCs (Figure 5). Our retinal whole mounts show an average of 844 ± 78 (*n* = 10) or 841 ± 110 (*n* = 10) ONs-αRGCs per retina, labeled with SMI32^+^Cal^+^Tbr2^+^ or OPN^+^Cal^+^Tbr2^+^, respectively, representing approximately 36% of all αRGCs (1.9% of the total RGC population; Figure 5). To ensure that all ONs-αRGCs expressed both SMI32 and OPN, we counted the number of SMI32^+^Cal^+^OPN^+^ RGCs which was 836 ± 81 (*n* = 10), comparable to previous counts (Schmidt et al., 2014; Krieger et al., 2017).

**Figure 6:**
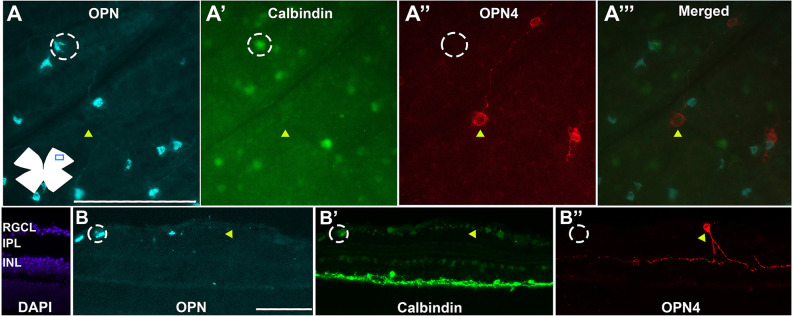
ONs-αRGCs are not immunodetected against melanopsin. Photomicrographs of flat-mounted retinas **(A–A^”’^)** or radial retinal sections **(B–B^”^)** immunodetected against the αRGC pan-marker OPN **(A,B)** in blue, Calbindin **(A’,B’)** in green and OPN4 **(A”,B”)** in red (merged in **A^”’^**) in the same area of the retina. White circles represent cells positive for OPN^+^ and Calbindin^+^ but negative for OPN4 (ONs-αRGCs/M4ipRGCs) and yellow arrowheads represent cells positive for OPN4 but negative for OPN and Calbindin (M1-M3-ipRGCs). Scale bar: 100 μm.

The ONs-αRGCs are intrinsically photosensitive and are classified as M4-ipRGCs (Schmidt et al., 2014; Lee and Schmidt, 2018; Sonoda et al., 2020). However, despite being a subtype of ipRGCs, these cells were not detected with the anti-melanopsin (OPN4) antibody (Figure 6); indeed, all cells that were OPN^+^Cal^+^ were not OPN4^+^, and no OPN4^+^ cells were OPN^+^ or Cal^+^, as shown in Figure 6. Therefore, with the immunolabeling techniques used in the present studies, the OPN4 antibody does not detect M4-ipRGCs. OPN4 antibody was able to immunodetect an average total number of 1,082 ± 99 cells per retina (*n* = 10), which probably corresponds to the M1–M3 subtypes and represents approximately 2.5% of all RGCs. This is consistent with previous findings in this laboratory (Valiente-Soriano et al., 2014).

In retinas analyzed in radial sections, the population of ONs-αRGCs was distributed throughout the retina, with higher densities in the temporal area (Figures 5B,D), a feature that is more evident in its topographical representation (Figure 4C). This color map shows that these cells are present throughout the retina, with a higher density in the temporal hemiretina and a lower presence in the central-nasal region (Figure 4C).

#### ONtransient-αRGCs

**Figure 7:**
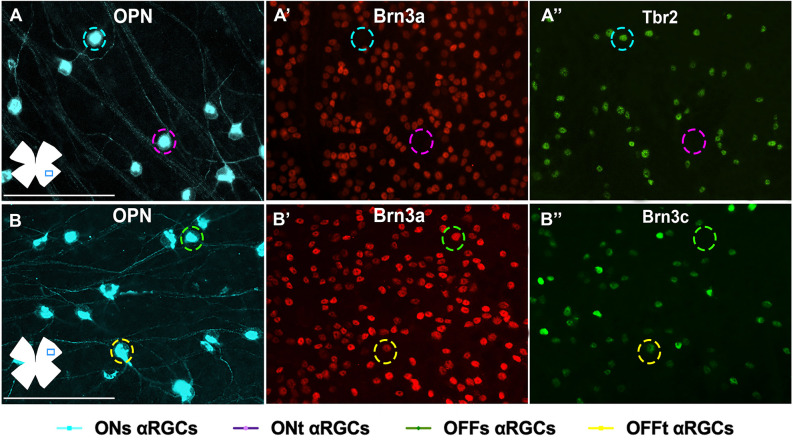
Immunohistochemical identification of αRGC types in flat-mounted retinas. Magnifications of flat-mounted retinas immunodetected against OPN, Brn3a, and Tbr2 **(A–A^”^)** to identify ONs-αRGCs (OPN^+^Brn3a^−^Tbr2^+^, blue traced circle), and ONt-αRGCs (OPN^+^Brn3a^−^Tbr2^−^, pink traced circle) and against OPN, Brn3a, and Brn3c **(B–B”)** to identify OFFs-αRGCs (OPN^+^Brn3a^+^Brn3c^−^, green traced circle) and OFFt-αRGCs (OPN^+^Brn3a^+^Brn3c^+^, yellow traced circle). Scale bar: 100 μm.

**Figure 8:**
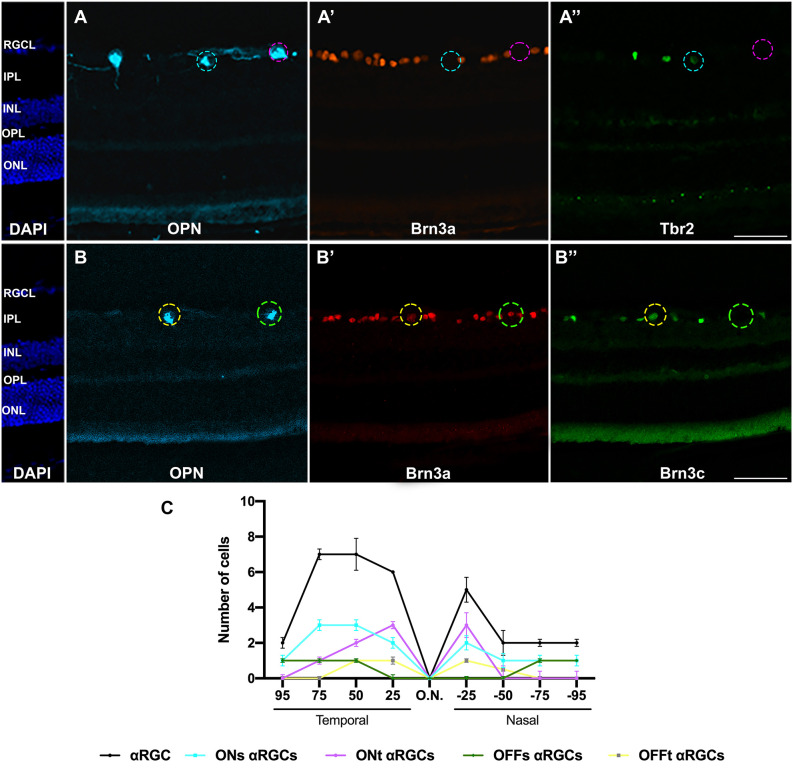
Immunohistochemical identification of αRGCs types in radial retinal sections. Magnifications of retinal sections immunodetected against OPN, Brn3a, and Tbr2 **(A–A”)** to identify ONs-αRGCs (OPN^+^Brn3a^−^Tbr2^+^, blue traced circle) and ONt-αRGCs (OPN^+^Brn3a^−^Tbr2^−^, pink traced circle) and against OPN, Brn3a, and Brn3c **(B–B”)** to identify OFFs-αRGCs (OPN^+^Brn3a^+^Brn3c^−^, green traced circle) and OFFt-αRGCs (OPN^+^Brn3a^+^Brn3c^+^, yellow traced circle). **(C)** Graph illustrating manual counting of αRGCs and its subtypes in different retinal regions, from 25-50-75-90% temporal to 25-50-75-90% nasal. Scale bar: 100 μm.

The ONt-αRGC population was identified as OPN^+^ cells that expressed neither Tbr2 nor Brn3a (Figures 7, 8). This subpopulation has an average of 797 ± 146 (*n* = 10) cells per retina, representing approximately 34% of all αRGCs (1.8% of total RGCs; Figure 4). The analysis of ONt-αRGCs in radial retinal sections shows a greater presence in the central regions of the retina with low presence in the temporal periphery and few to none in the nasal periphery (Figure 8). This characteristic is observed in more detail in their whole mount topographical representation which shows a higher density of ONt-αRGCs in the central-middle retinal area with few cells located in the retinal periphery (Figure 4D).

#### OFFsustained-αRGCs

To identify the OFFs-αRGC subpopulation, OPN^+^ cells were colocalized with the Brn3a antibody, which is expressed in this cell subtype (Krieger et al., 2017; Figures 7, 8). The average total number of OFFs-αRGCs per retina was 363 ± 50 (*n* = 10), representing approximately 16% of all αRGCs (0.8% of the total population of RGCs; Figure 4). Analysis of OFFs-αRGCs in radial retinal sections showed that these cells are less abundant in central retinal regions, maintaining their presence in the middle-peripheral retina (Figure 8). Its topographical whole mount map confirms this organization, showing a higher density in the supero-temporal peripheral region of the retina (Figure 4E).

#### OFFtransient-αRGCs

This subtype of αRGCs was identified using similar immunohistochemical techniques used to identify OFFs-αRGCs. But, in this case, OPN^+^ (all αRGCs), Brn3a^+^ (all OFF-αRGCs), and Brn3c^+^ were identified as OFFt-αRGCs (Krieger et al., 2017; Figures 7, 8). The proportion of OFFt-αRGCs [376 ± 53 (*n* = 10)], is almost identical to that of OFFs-αRGCs, representing approximately 16% of all αRGCs (0.8% of the total RGC population; Figure 4), although their distribution is slightly different as OFFt-αRGCs are preferentially distributed in the middle retina, mostly in the infero-temporal region (Figure 4F).

## Discussion

In this work, we have further characterized αRGCs and their subtypes. We have analyzed their total numbers and topographical distribution within the adult pigmented mouse retina. Although αRGCs have been extensively studied in the literature and are readily identifiable by their morphology, functional responses, and arrangement of their dendritic arbor (Cleland et al., 1975; Dreher et al., 1985; Krieger et al., 2017; Bae et al., 2018), to our knowledge, there is no consensus on the total numbers and distribution of this population and its subtypes in the mouse retina.

### Total αRGC population in the pigmented mouse retina

In this work, we have used immunohistochemical techniques to identify αRGCS and its subtypes which are classified depending on the type of central response (ON-OFF) and whether this response is sustained or transient (ONs-αRGCs, corresponding to the population of M4-ipRGC, ONt-αRGCs, OFFs-αRGCs, and OFFt-αRGCs) in the pigmented mouse retina. Following previous studies, SMI32 or OPN antibodies were used to identify the total αRGC population (Krieger et al., 2017; Lee and Schmidt, 2018; Tan et al., 2022). These antibodies are expressed throughout the αRGC cell body, labeling somas, dendrites, and axons, enabling these cells to be localized throughout the retina. However, OPN labels less intensely the axons and this facilitates the identification of the somas located near the optic nerve head (Figure 2).

The mean population of SMI32- or OPN-labeled RGCs was very similar in mouse retinas (2,252 ± 306 and 2,315 ± 175, respectively). These data are slightly higher than those reported by Schmidt et al. (2014) which counted a total of 1,613 αRGCs per mouse retina. Another study showed the mean density of OPN^+^RGCs in the mouse retina with a mean density of ≈250 cells/mm^2^ (Honda et al., 2019), slightly higher than our results (≈160 cells/mm^2^). These discrepancies may be explained because densities were estimated by taking photographs of the middle regions of the retina, omitting the central regions close to the optic nerve and the peripheral regions. This may overestimate the result as these areas, as shown in the distribution graph (Figure 4B), clearly have a lower density of cells. Other studies in rat retina show lower density data (60–130 cells/mm^2^) for SMI32^+^ (Tan et al., 2022), OPN^+^ (Gallego-Ortega et al., 2021) or SMI32^+^OPN^+^ (Mayer et al., 2018) αRGCs than our data, although this may be because the RGC density in rat retina is lower than in mouse retina (Nadal-Nicolás et al., 2018). Tan et al. (2022) indicate a limitation in counting SMI32^+^αRGCs in flat retinas due to the intense axon labeling that hinders the visualization of somas in the region close to the optic nerve head. We also experienced this difficulty in our study and therefore we performed the study of αRGC subtypes with the OPN antibody. Our data show that 5.08% of all RGCs are SMI32^+^αRGCs and 5.23% are OPN^+^αRGCs. These data differ slightly from those reported by Schmidt et al. (2014) (3.7% SMI32^+^αRGCs) and Honda et al. (2019) (≈8.9% OPN^+^αRGCs). A possible reason for these discrepancies may be related to the fact that we count the entire population of RGC labeled cells over the entire retina whereas the other studies sample small regions of the retina.

However, there is more consensus on the description of the topographical distribution of αRGCs in the retina. The color maps show that αRGCs are located throughout the retina although they acquire a higher density in the temporal region (Figures 4A,B), suggesting, as previously indicated by Bleckert et al. (2014), that they have greater activity in the central visual field. These results are also consistent with previously documented distribution (Dreher et al., 1985; Peichl, 1989; Sonoda et al., 2020; Tan et al., 2022) except in the work of Schmidt et al. (2014), which suggests a more homogeneous distribution throughout the retina.

### Population and distribution of αRGC subtypes

In this work, we studied the population and distribution of αRGCs subtypes using immunohistochemical techniques. Previous studies had identified three types of αRGCs, ONs-αRGCs, OFFs-αRGCs, and OFFt-αRGCs (Cleland et al., 1975; Pang et al., 2003; Van Wyk et al., 2009) until Krieger et al. (2017) characterized physiologically and molecularly the fourth type of αRGCs (ONt-αRGC) in the mouse retina.

The most studied αRGC subtype in the literature is the ONs-αRGC population because it constitutes the M4-ipRGCs, a subtype of ipRGCs that is not detectable with the classical anti-melanopsin antibody (OPN4) that only detects M1, M2, and M3 ipRGCs (Estevez et al., 2012; Schmidt et al., 2014; Abud et al., 2017; Berg et al., 2019; Honda et al., 2019; Sonoda et al., 2020), an observation that we have confirmed in the present studies, there were no ONs-αRGC stained with OPN4 antibodies (Figure 6). In this work, we have designed an immunodetection protocol to detect the ONs-αRGC (M4-ipRGC) population using the αRGCs pan-markers SMI32 or OPN and calbindin (expressed in ONs-αRGCs and amacrine cells; Krieger et al., 2017) or Tbr2, a key transcriptional regulator for the development and maintenance of ipRGCs (expressed in ipRGCs and GABA-displaced amacrine cells; Berg et al., 2019; Tran et al., 2019; Chen et al., 2021). Therefore, these protocols indicate that this αRGC subtype expresses these four proteins, making them easily identifiable. In this work we have detected an average of ~840 ONs-αRGCs (M4-ipRGCs) per mouse retina, representing approximately 36% of all αRGCs (1.9% of total RGCs), and this is in agreement with a recent study (Gao et al., 2022) reporting a density of 52 ONs-αRGCs/mm^2^. Our data, however, differ slightly from those published in previous work in which the percentage of ONs-αRGCs (M4-ipRGCs) was slightly lower. Krieger et al. (2017) reported that approximately 27% of αRGCs were ONs-αRGCs. Another study documented that ON-αRGCs (without differentiating between sustained or transient type) represented approximately 50% of αRGCs (Schmidt et al., 2014), while our data estimates the total percentage of ON-αRGCs of approximately 70% of all αRGCs. These differences could be explained because the above-mentioned studies estimate total RGC numbers based on retinal densities obtained from sampling specific retinal regions, whereas our numbers are based on total counts over full photomontages. Sampling retinal regions may lead to calculation errors because RGCs are not distributed homogenously across the retina, as shown before for the total RGC population of the mice retina (Salinas-Navarro et al., 2009; Galindo-Romero et al., 2011; Ortin-Martinez et al., 2014) and the αRGC population in this work.

Regarding the population of ONt-αRGCs, our data show a 34% proportion (797 ± 146 per retina) of the total number of αRGCs and this is in agreement with Krieger et al. (2017) reporting that 25% of αRGCs expressed neither calbindin, Brn3a nor Brn3c and are therefore classified as ONt-αRGCs. Concerning the OFF-αRGCs population, our data show that they constitute 32% of the αRGCs, slightly less than the 48% documented by Krieger et al. (2017) or the 50% documented by Schmidt et al. (2014). Our data do agree with data provided by Krieger et al. (2017) on the proportion of OFF-αRGCs of sustained or transient response that divides at exactly 50% (48% of OFFs-αRGCs and 52% of OFFt-αRGCs in the study of Krieger et al. (2017). Wang et al. (2021), using the molecular marker Kcnip2 to identify OFFt-αRGCs, document that these show a density recovery profile typical of a distinctive type of RGCs and that their densities accounted for approximately 1.5% of the total RGC population, a slightly larger percentage than that found in our present study.

Although OFF-αRGCs are less numerous than ON-αRGCs, their distribution parallels the two types depending on the type of sustained or transient response (Figure 4). Sustained-response αRGCs are mostly distributed in the superior and temporal quadrants of the retina, with a higher density in the mid-peripheral part of the retina (Figures 4C,E). However, transient-response αRGCs are preferentially located in the central-temporal region of the retina with a lower presence in the peripheral region (Figures 4D,F). While the underlying functionality of this distribution of αRGCs, located throughout the retina, with a higher density in the central-peripheral temporal area (especially sustained response αRGCs), is unknown, SMI32^+^RGCs have been previously described to have asymmetric arbors of normal or slightly enlarged size, similar to those described in melanopsin cells, but have no dendritic contact between homotypic cells, although dendritic contact is necessary for cell-cell spacing and for controlling the size of dendritic arbors (Lin et al., 2004; Tan et al., 2022). This network of extensive dendritic fields suggests that these cells may have a functional action throughout the visual field, although they are most prominent in the central visual field.

### Limitations of the present studies and concluding remarks

Our present work is limited to the αRGCs identified with immunohistochemistry using a combination of antibodies known to be expressed by different α-RGCs subtypes. However, we did not use functional techniques to determine their response to light, nor did we use morphological techniques to measure their soma and dendritic field sizes, or study their laminar distribution within the inner plexiform layer, and thus, perhaps it would be more appropriate to refer to these cells as α-like RGCs (Bleckert et al., 2014; Ou et al., 2016; Tan et al., 2022). The present studies preclude reliable identification of all αRGCs and thus we cannot state that all cells immunodetected in our present work are those with the largest soma in the RGC layer, e.g., mouse retinal neurons in the Eyewire Digital Museum^1^ (Bae et al., 2018) as big as the OFFt-αRGCs, neither can we state that our study includes the miniature αRGCs (Baden et al., 2016).

Here, using a simple protocol to identify each of the αRGC subtypes, we further characterize the topographic distribution and the total numbers of each of these subtypes in the mouse retina. This approach may be relevant in future work on the study of the response of each cell type to a retinal pathology or a specific treatment.

## Data Availability Statement

The raw data supporting the conclusions of this article will be made available by the authors, without undue reservation.

## Ethics Statement

The animal study was reviewed and approved by A13170110, Comité Ético de Experimentación Animal (CEEA) de la Universidad de Murcia and Consejería de Agua, Agricultura, Ganadería, Pesca y Medio Ambiente de la Universidad de Murcia. Written informed consent was obtained from the owners for the participation of their animals in this study.

## Author Contributions

AG-O and MV-S: conceptualization. AG-O, MN-M, and JD: methodology and formal analysis. AG-O: investigation. MA-T, PV, and MV-S: resources and funding acquisition. AG-O, FV-S, and MV-S: writing—original draft preparation, writing—review and editing. All authors contributed to the article and approved the submitted version.

## Funding

This research was funded by PID2019-106498GB-I00 funded by Ministerio de Ciencia, Innovación y Universidades (MCIN)/AEI/ 10.13039/501100011033 to MV-S and MA-T; (RetiBrain) RED2018-102499-T to MV-S; and by the Instituto de Salud Carlos III and co-funded with the European Regional Development Fund (ERDF) within the “Plan Estatal de Investigación Científica y Técnica y de Innovación 2017–2020” (FIS/PI 18-00754) to PV.

## Footnotes

^1^ http://museum.eyewire.org
